# Relationship between transcranial magnetic stimulation markers of motor control and clinical recovery in obsessive compulsive disorder/Gilles de la Tourette syndrome: a proof of concept case study

**DOI:** 10.3389/fpsyt.2024.1307344

**Published:** 2024-01-17

**Authors:** Caroline Quoilin, Fostine Chaise, Julie Duque, Philippe de Timary

**Affiliations:** ^1^CoActions Lab, Institute of Neuroscience, Université catholique de Louvain, Brussels, Belgium; ^2^Department of Adult Psychiatry, Cliniques universitaires Saint-Luc, Brussels, Belgium

**Keywords:** obsessive compulsive disorder, Gilles de la Tourette syndrome, movement preparation, transcranial magnetic stimulation, motor excitability, inhibitory control, neurodevelopmental disorders

## Abstract

**Background:**

Obsessive compulsive disorder (OCD) and Gilles de la Tourette syndrome (GTS) are neurodevelopmental disorders characterized by difficulties in controlling intrusive thoughts (obsessions) and undesired actions (tics), respectively. Both conditions have been associated with abnormal inhibition but a tangible deficit of inhibitory control abilities is controversial in GTS.

**Methods:**

Here, we examined a 25 years-old male patient with severe OCD symptoms and a mild form of GTS, where impairments in motor control were central. Transcranial magnetic stimulation (TMS) was applied over the primary motor cortex (M1) to elicit motor-evoked potentials (MEPs) during four experimental sessions, allowing us to assess the excitability of motor intracortical circuitry at rest as well as the degree of MEP suppression during action preparation, a phenomenon thought to regulate movement initiation.

**Results:**

When tested for the first time, the patient presented a decent level of MEP suppression during action preparation, but he exhibited a lack of intracortical inhibition at rest, as evidenced by reduced short-interval intracortical inhibition (SICI) and long-interval intracortical inhibition (LICI). Interestingly, the patient’s symptomatology drastically improved over the course of the sessions (reduced obsessions and tics), coinciding with feedback given on his good motor control abilities. These changes were reflected in the TMS measurements, with a significant strengthening of intracortical inhibition (SICI and LICI more pronounced than previously) and a more selective tuning of MEPs during action preparation; MEPs became even more suppressed, or selectively facilitated depending on the behavioral condition in which they we probed.

**Conclusion:**

This study highlights the importance of better understanding motor inhibitory mechanisms in neurodevelopmental disorders and suggests a biofeedback approach as a potential novel treatment.

## 1 Introduction

Obsessive compulsive disorder (OCD) is a neurodevelopmental behavioral and mental syndrome in which patients develop ritual behaviors (compulsions) to escape intense distress generated by uncontrolled intrusive thoughts (obsessions) (1). The most common obsessions are the fear of contamination and compulsive washing, the obsessions with order and symmetry, the doubts about recent actions that require verification, and the fear of not being able to control impulses (e.g., fear of acting blasphemously or fear of harming others or themselves). Gilles de la Tourette syndrome (GTS) is another neurodevelopmental behavioral and mental disorder characterized by an impossibility to control undesired actions (1), resulting in sudden, rapid, recurrent and non-rhythmic motor and vocal tics (2). Tics in GTS are generally preceded by a premonitory urge, which refers to a subjective feeling of physical tension or pressure, that cannot be controlled and is temporally relieved following tic expression (3–6). The presence of premonitory urges in OCD is more controversial (7–9). GTS and OCD are not rare conditions, with epidemiological data indicating that the prevalence is around 1% for both; GTS is 3 or 4 times more frequent for men than women (10, 11), while OCD is slightly more frequent for women (12). In about 90% of the cases, GTS present comorbid conditions, the most frequent being OCD and attention deficit hyperactivity disorder (ADHD), but anxiety, affective and impulse control disorders are also frequently reported (10, 11, 13).

The clinical case developed in this paper is that of a patient who presents both severe OCD symptoms and a mild form of GTS, but where the entire pathology is characterized by difficulties with control of movements or impulsions. Tics in GTS are generally considered as the consequence of dysfunctional cortico-striato-thalamo-cortical circuits, in which abnormally active subsets of striatal neurons induce a disinhibition of thalamo-cortical projections, which in turn results in an enhanced cortical excitability in motor areas, such as the primary motor cortex (M1) or the supplementary motor area (SMA) (14–16). In particular, GTS has been associated with an alteration in GABAergic systems. As such, postmortem analyses (17) and positron emission tomography imaging (18) have highlighted a widespread decrease in the density of GABA-A receptors. Furthermore, several paired-pulse transcranial magnetic stimulation (TMS) studies have shown that short-interval intracortical inhibition (SICI), a phenomenon depending on GABA-A receptors activity (19), is significantly reduced within M1 in GTS patients (20, 21), with the strength of the defect being related to tic severity (22, 23). Importantly, similar observations have been made in relation to OCD (24), with many TMS studies clearly showing alterations of motor inhibition circuits, including SICI (25–27), although findings have not always been consistent (28, 29).

Importantly, the literature in GTS is not completely in favor of a deficit in inhibitory control, as recently reviewed (30, 31). Most of GTS patients are able to temporarily suppress tics on demand (32, 33), leading some researchers to rather suggest that these patients would have enhanced motor inhibitory abilities, trained by the frequent need to inhibit tics (34, 35). By using functional magnetic resonance imaging to contrast brain activity during free ticking and voluntary tic inhibition states, previous works have indeed shown that tic inhibition is a cognitive process that recruits frontal areas (36–38). In addition, a past single-pulse TMS study has related phasic voluntary tic inhibition to decreased excitability of the motor corticospinal pathway (32). When applied over M1, single-pulse TMS elicits motor-evoked potentials (MEPs) in targeted contralateral muscles, with the amplitude of these MEPs reflecting the excitability of the corticospinal pathway at the time of stimulation (39). Interestingly, Ganos et al. (32) showed that the amplitude of MEPs is reduced in GTS patients trying to control their tics, suggesting that the motor output can be voluntarily inhibited to prevent tics from reaching the threshold for expression. Relatedly, two past studies have used the stop-signal task to assess reactive and proactive inhibitory control in drug naïve children/adolescents affected by GTS, OCD, or both, and the findings are not consistent with an alteration of inhibitory control in GTS but well so in OCD. Specifically, Mancini et al. (40) reported that both reactive and proactive inhibition scale with the severity of OCD symptoms but not with that of tic symptoms. Results from Mirabella et al. (41) further show that drug naïve GTS patients do not exhibit impaired inhibitory control while OCD patients display both impaired inhibitory control and altered underlying brain regions. Hence, the occurrence of inhibitory control deficits in patients displaying both syndromes, such as the clinical case presented in this paper, is more likely related to their OCD than to GTS (30).

While the studies highlighted above have assessed inhibitory control at a behavioral level, a different approach is to consider inhibitory changes occurring in motor neural activity during action preparation (39, 42). There is strong evidence that preparing a movement involves a suppression of activity in the corticospinal motor output pathway. This suppression is typically probed by applying single-pulse TMS over M1, allowing to record MEPs, during variants of instructed-delay choice reaction time (RT) tasks (42–44). Such tasks require participants to choose a response based on a preparatory cue (for instance, a movement of the left or right index finger), and to withhold this response until the onset of an imperative signal. When probed before the imperative, MEPs elicited in task-relevant and task-irrelevant muscles of both hands are strongly suppressed relative to resting conditions (42, 45–47). This drastic suppression of motor excitability is thought to help inhibit premature or inappropriate responses and, more generally, to ensure some sort of inhibitory control during action preparation [(42, 43); but see also (48–51) for an alternative hypothesis]. This idea comes from the observation that motor suppression is deeper when movement preparation entails overcoming sensory conflict (52, 53). In addition, MEP suppression is weaker in impulsive individuals such as pathological gamblers (54) or alcohol-dependent patients (55–58). Interestingly, in the latter group, the deficit in motor suppression is related to the risk of relapse and to the neurotoxic effects of alcohol in prefrontal areas (56, 58). Importantly, as action preparation also entails excitatory processes to prepare the motor system for the forthcoming action, the MEP suppression can be weaker in the selected effector (i.e., the muscle involved in the response), especially when the tendency to act is particularly strong (59–61). As a result, whereas the tendency to act can be estimated based on MEPs in selected effectors, MEPs probed in task-irrelevant muscles of the non-selected hand are likely to represent the purest probe of inhibitory influences related to inhibitory control, spared from the excitatory drive associated with the planned response (45).

In the present study, we used TMS over M1 to assess motor inhibition, by considering both (1) the excitability of motor intracortical circuits at rest and (2) the degree of motor suppression during action preparation, as a physiological probe of inhibitory control, in a 25 years old male patient suffering from difficulties in motor control inhibition while suffering from a severe OCD and GTS. Notably, our protocol focused on motor physiology and did not involve any measure of inhibitory control at a behavioral level; the RT task we used is not designed to provide this type of measurement (58). Accordingly, we focused on MEP measurements obtained at rest and during action preparation. Paired-pulse TMS was used at rest to evaluate the strength of intracortical circuits within M1, including SICI but also long-interval intracortical inhibition (LICI) and intracortical facilitation (ICF). In addition, single-pulse TMS was applied over M1 to elicit MEPs in task-relevant and task-irrelevant muscles during action preparation, when the patient was performing an instructed-delay choice RT task, in order to assess the level of preparatory changes in corticospinal excitability. Importantly, the patient was tested four times, in a period during which his symptomatology evolved drastically, allowing us to investigate whether motor excitability at rest and during action preparation changed in parallel with clinical expression. For comparative purposes, we also include MEP data from a group of healthy controls (*n* = 17) performing the same instructed-delay choice RT task.

## 2 Materials and methods

### 2.1 Case presentation and time course of the study

#### 2.1.1 General overview

The patient is a right-handed man who was 25 years old at the time of the four TMS assessments that were conducted from February to April 2021 as part of this study. He had been admitted to the psychiatric unit of the Cliniques Universitaires Saint-Luc (CUSL, UCLouvain, Brussels) about 10 months earlier (in June 2020), when aged 24, to consult with Prof. Philippe de Timary, with a diagnosis of a severe OCD. The patient reported that the OCD originally developed in 2012 when he was 16 years old, together with a major depressive episode, at a time when the family was facing intense difficulties that generated an important stress for the patient. Back then, his main fear was of not being able to control non-adapted, unmentionable impulsions, a state that could be qualified as a phobia of impulsions, that degenerated into obsessions. To avoid these thoughts, he had developed various types of compulsions. Not surprisingly, the patient presented with strong difficulties to finish secondary school but finally obtained graduation when he was 18. Thereafter, he attempted to start university several times but was unable to attend lectures.

**Figure 1:**
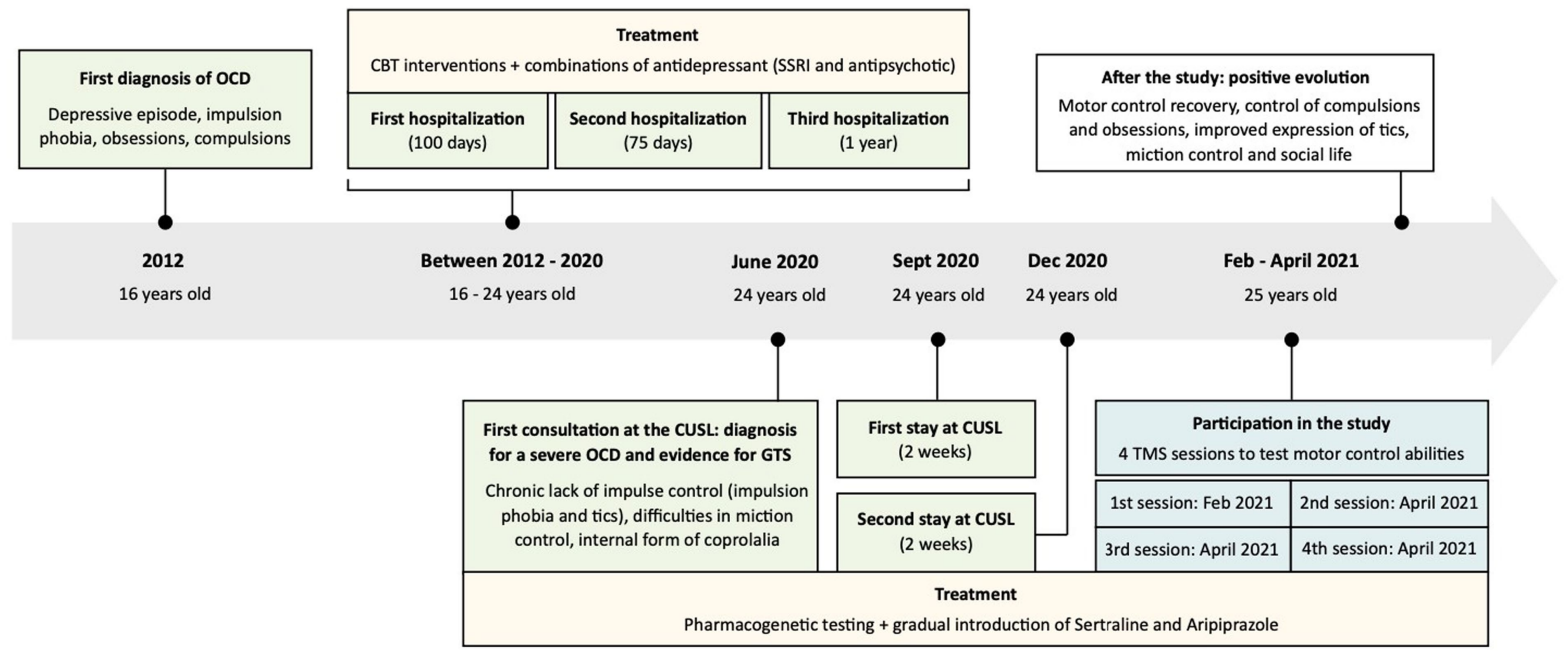
Timeline describing the pathology and patient care. Past medical history: episodes of externalization during childhood (strong angers and break objects), one episode of obsessive compulsive disorder (OCD) reported around 5 years-old (symptoms recovered with the support of a psychotherapist), excelled at school, enjoyed social interactions and involved in sports and scouting activities during adolescence. First diagnosis of OCD in 2012, followed by three hospitalizations in psychiatry and the implementation of a specific treatment [cognitive behavioral therapy (CBT), Selective Serotonin Reuptake Inhibitor (SSRI) and antipsychotic]. First consultation at the Cliniques Universitaires Saint-Luc (CUSL) and diagnosis for a severe OCD and evidence for Gilles de la Tourette Syndrome (GTS) in June 2020: severe OCD with strong consequences on the patient’s life, tics (essentially blinking and facial spasms), internal form of coprolalia, presence of a chronic motor tic disorder, with impulsion phobia and tics, difficulties in miction control. The diagnosis was followed by the implementation of a new treatment (Sertaline and Aripiprazole) and two new hospitalizations at CUSL. Participation in the study: test his motor control abilities with four transcranial magnetic stimulation (TMS) sessions (the patient was still suffering from severe OCD). After the study: positive evolution with a shift in behavior. Observation of motor control recovery, improvements in the expression of tics, a control over compulsions and obsessions, improvements in miction control and in social life.

Hence, when the patient was tested in our study, in 2021, he had been suffering from a severe OCD pathology for 9 years, a state that profoundly altered his evolution. He had already been hospitalized three times for prolonged stays in psychiatry (100 days, 75 days, 1 year; see Figure 1 for a timeline describing the pathology and patient care). During these stays (and back home), the patient had been receiving specific OCD targeting cognitive behavioral therapy (CBT) interventions, as well as several combinations of antidepressant (high doses of SSRI and antipsychotic medications), to try to improve the situation. Yet, the patient had remained severely affected by the disorder. Before the age of 16, it appears that the patient was a brilliant pupil, enjoying social interactions and having strong bonds with his friends; he was involved in sports and scouting activities. Note though that the patient had already suffered an episode of OCD once, around the age of 5, in a period of intense stress, but symptoms had recovered with the support of a psychotherapist. Moreover, the family and patient also reported episodes of impulsive externalization during childhood, where he would get into clastic wrath and break objects.

#### 2.1.2 Clinical observations at the time of the first consultation at CUSL: evidence for OCD and GTS

The main symptoms were that of a severe OCD with strong consequences on the patient’s life. He was totally withdrawn within his mother’s house from which he would hardly get out. Going out or meeting people would lead to intense anxiety. Meeting others would be followed by mental automatisms and hours of psychic rituals, to “clean up his mind” from interferences with others. For these reasons, going to an appointment would lead to deep anxiety and need a very long preparation. Frequently, he would not be able to get to a planned meeting because preparation rituals would take too much time.

Besides the OCD, which was the main complaint of the patient, we observed that he also presented with tics that expressed essentially as blinking and facial spasms, that he described as more frequent in periods of stress. Discussing with the parents, we learned that there was a history of tics within the family of the father. Furthermore, the patient would not exhibit vocalizations often observed in complete GTS but he would report with frequent internal insulting that could be considered as an internal form of coprolalia he could not control for. Although not typical of a GTS, we considered that the patient presented a chronic motor tic disorder according to DSM-5 criteria (1). Besides the impulsion phobia and tics, that both suggested a difficulty of the patient for inhibitory control, he had also recently developed difficulties in miction control, that presented as “accidents” and emergencies, where he would start urinating without deciding it, leading to wetting his pants.

In relation with his situation of a severe OCD pathology, after meeting the patient in June 2020, we planned with him a pharmacogenetic testing of CYP2B6 and CYP2C19 genes (62) to ensure correct choice of medication and thereafter started gradually the introduction of increasing doses of Sertraline, an SSRI, and Aripiprazole, an atypical antipsychotic with D2 partial agonist properties, that are generally considered as potent medications in both OCD (63) and GTS (64). Sertraline and Aripiprazole were progressively raised to 300 and 15 mg/day respectively, and had reached a steady state in December 2020, meaning that the medications were stable when the patient was tested in the context of this study.

#### 2.1.3 Patient evolution after the first consultation at CUSL and during the TMS motor testings in our study

The patient realized a first two-week stay at CUSL, in September 2020. The OCD was severe and the patient would remain for the whole stay in his bedroom, avoiding contacts with other patients, to prevent the development of prolonged compulsive episodes, that he described as necessary to “clean up his mind” from interference with others. He was not taking care of himself and would not accept taking a shower or changing his clothing except twice during his stay where it took him 3 to 4 h to take the shower.

During a second 2 weeks stay, in December 2020, the OCD was nearly as severe, but the patient was discussing about planning to join in the common activities of the treatment group. There was a slight improvement in the level of anxiety. However, he was unable to start any activity, spending all the time in his bedroom except 1 day where he went to look at the timetable of the activities.

In February 2021, because of the existence of a history of impulse control difficulties during childhood, because in the OCD, the main obsession leading to compulsions was the fear of not being able to control for motor impulsions, because of the existence of tics, and of difficulties to control miction, we decided to test thoroughly the motor control abilities of the patient using TMS, as we had previously done in patients suffering from addiction (58), following the methodology described below. The TMS motor control testings were performed four times, first in February 2021 and then on three occasions separated by a week interval during a hospital stay in April 2021. Written informed consent was given by the patient before each session, following a protocol approved by the Biomedical Ethics Committee of the Saint-Luc University Hospital, Université catholique de Louvain (B403201836840; 2018/22MAI/219).

The patient showed to be very interested by the approach aimed at testing his motor circuits and control abilities: we observed significant changes in his behavior over the course of the sessions, especially after the second session. Intriguingly, this shift coincided with a long discussion the patient had with the experimenter (CQ) following this session, aiming at giving him some feedback on prior measurements. Indeed, CQ had already analyzed the data obtained during the first session, and observed that the degree of MEP suppression during action preparation, a physiological marker of inhibitory processes, was normal in the patient (see Results section), suggesting good inhibitory control abilities. In particular, she insisted on the fact that, when required, he had the appropriate resources to exert control over his behavior. After this discussion, there was a complete shift in behavior where the patient suddenly decided to quit his room to join into activities of the group of patients. From this date on, the change in behavior was complete: he would not use his bedroom as a hiding place anymore, he started taking showers and changing his clothes every day. He also described that he had a better control over his compulsions and that time spent on obsessions was largely decreased. In view of the behavioral and motor control improvements, a fourth testing was performed during the same hospital stay, to evaluate the persistence of the motor control recovery. This last testing confirmed what was observed during the third session.

Since then, the patient has continued his positive evolution, somehow mastering his compulsions and obsessions that gradually took less and less space in his life. Obsessions scores, as expressed with the Yale-Brown Obsessive Compulsive Scale (YBOCS), were around 35 before treatment (September to December 2020) and are currently of 8. There was also a large improvement in the expression of tics, evaluated with the Yale Global Tic Severity Scale (YGTSS), and that are currently very rare (YGTSS score evolved from 72 to 6). Interestingly, with the support of a specialized physiotherapist, he could also progressively improve his control of the bladder that is currently normalized. Collectively, he has regained a much better “motor” control over thoughts and behaviors and escape the “freezing” pattern he was involved in. The patient could progressively start university courses, spend time with friends, participate to parties and even join in a flat shared with roommates. In follow up consultations, he is accompanied in resocialization processes that is about as important as the success in university courses.

### 2.2 Healthy participants

The data from 17 right-handed healthy volunteers (8 women; mean age = 22.6 ± 2.06 years old), in which changes in corticospinal excitability during action preparation were previously evaluated, were used to compare the patient data to a control group. All healthy participants had no history of neurological or psychiatric disorders. Those data were also used for another publication (45).

### 2.3 Experimental procedure

As detailed in the case study description, the patient underwent four similar TMS testing sessions. During each session, he sat on a chair with his arms semi-flexed and both hands resting palm-down on a table and was explicitly asked to stay still and keep his eyes open. Excitatory and inhibitory intracortical circuits within the dominant M1 were probed during the first two blocks (approximate duration of 4 min). Then, changes in corticospinal excitability during action preparation were assessed during the four subsequent blocks (approximate duration of 7 min per block). Here, measures were obtained in the left and the right hand on separate blocks, and the order of stimulation was counterbalanced across the sessions. A short break was given between each of these six blocks.

#### 2.3.1 Motor excitability at rest

**Figure 2:**
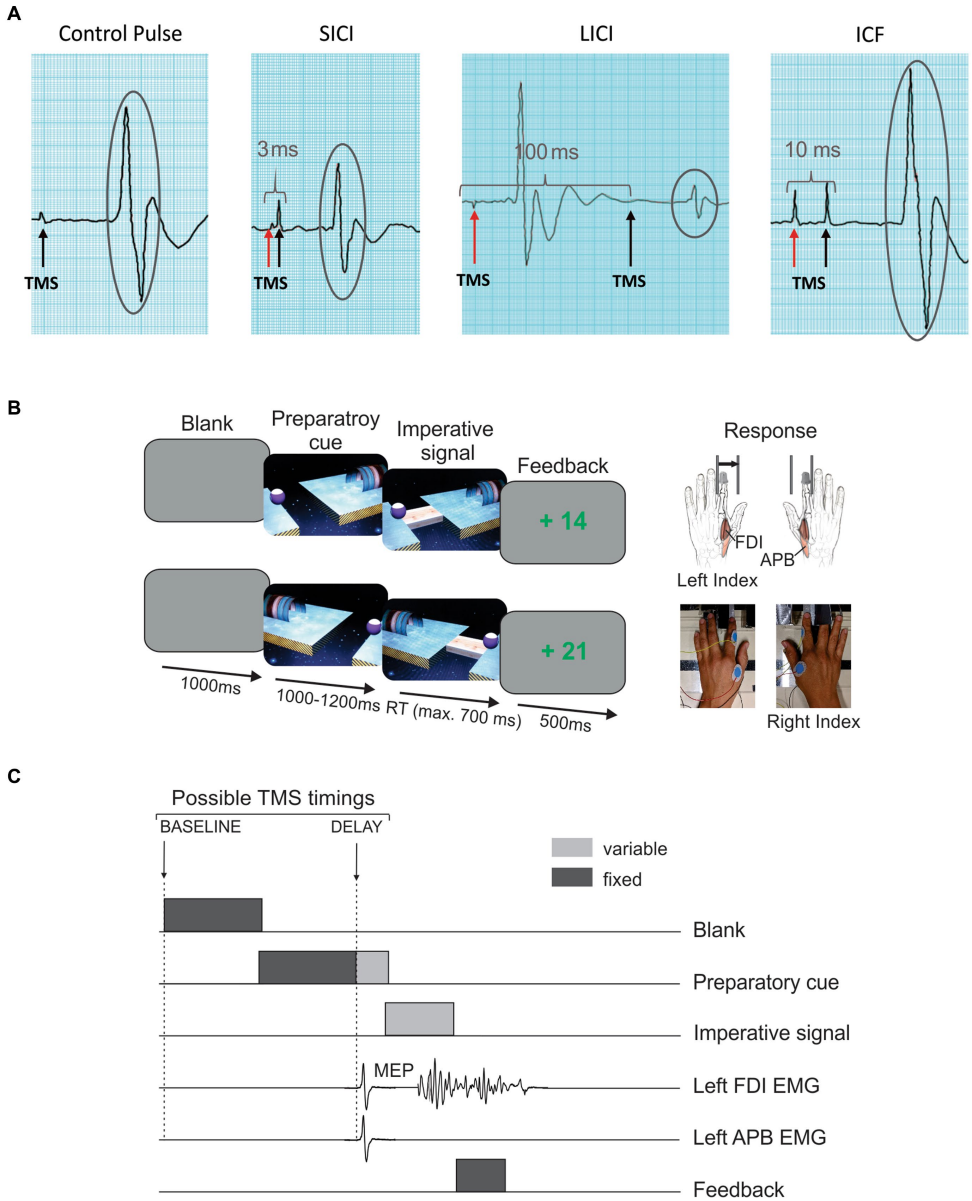
TMS session. **(A)** Measure of intracortical circuits. Paired-pulse TMS was used to assess short-interval intracortical inhibition (SICI), long-interval intracortical inhibition (LICI) and intracortical facilitation (ICF). To do so, the impact of a first conditioning stimulus (red arrow) on the motor-evoked potential (MEP; encircled in grey) elicited by a second test stimulus (black arrow) was evaluated. The amplitude of conditioned MEPs in each condition was then compared to the mean amplitude of MEPs elicited by a single, control pulse. **(B)** Rolling ball task. This instructed-delay choice reaction time task required to choose between an abduction movement of the left (upper trace) or right (lower trace) index finger depending on the position of a preparatory cue (i.e., the ball), and to withhold the response until the onset of an imperative signal (i.e., the bridge). Then, the response had to be released as fast as possible. Index finger responses were recorded using a home-made response device. **(C)** TMS timings. One single TMS pulse was delivered in each trial over the left or the right primary motor cortex (separate blocks) to elicit MEPs in the contralateral first dorsal interosseous (FDI) and abductor pollicis brevis (APB) at two possible timings: either at the onset of the blank screen (TMS_BASELINE_) or 950 ms after the onset of the preparatory cue (TMS_DELAY_).

Short-interval intracortical inhibition (SICI), long-interval intracortical inhibition (LICI) and intracortical facilitation (ICF) were assessed as an indirect measure of GABA-A (19), GABA-B (65) and NMDA (66) receptor-mediated neurotransmission within M1, respectively. To do so, we used a paired-pulse TMS paradigm (67–69), in which two magnetic stimuli are delivered through the same stimulating coil in order to evaluate the effect of the first (conditioning) stimulus on the second (test) stimulus (Figure 2A). The intensity of the conditioning stimulus was set either at 80% (SICI and ICF) or 120% (LICI) of the resting motor threshold (rMT), while the intensity of the test stimulus was always set at 120%. Four conditions were presented. In the control condition, the test stimulus was given alone. In the other conditions, the conditioning stimulus was given prior to the test stimulus at an inter-stimulus interval of 3 ms (SICI), 10 ms (ICF), or 100 ms (LICI). Overall, 16 trials of each paired-pulse condition and 24 trials of the control condition were recorded in a random order (72 trials in total, divided into 2 blocks of 36 trials). Then, each conditioned MEP (MEPs elicited by the test stimulus in the paired-pulse condition) was expressed in percentage of changes relative to the mean amplitude of the unconditioned MEPs (MEPs elicited in the control condition). Hence, a negative value reflects intracortical inhibition, while a positive value indicates intracortical facilitation.

#### 2.3.2 Motor excitability during action preparation

Changes in corticospinal excitability during action preparation were evaluated by applying single-pulse TMS over the left and right M1 (separate blocks) during an instructed-delay choice RT task implemented with Matlab 7.5 (MathWorks, Natick, Massachusetts, United States) using the Psychophysics Toolbox extensions (70, 71). This task consisted in a virtual “rolling ball game” previously used in other studies (50, 54, 72) and requiring participants to choose between responding with a left or a right index finger abduction according to the position of a preparatory cue that appeared on a computer screen (i.e., a left or right-side ball separated from a goal by a gap). Importantly, participants were asked to withhold their response until the onset of an imperative signal (i.e., a bridge connecting the ball and the goal) and to then respond as fast as possible. As such, they received the following instruction: “move the right finger if the ball is on the right side, or the left finger if the ball is on the left, as if you were kicking the ball, but do so only once the bridge appears, as fast as possible.” Responses were monitored using a response device developed in our laboratory (59); once a correct response was detected, the ball rolled over the bridge to reach the goal. By contrast, a response provided before the onset of the imperative caused the ball to fall into the gap.

The sequence of events of a typical trial is shown on Figure 2B. Each trial started with the presentation of a blank screen for 1,000 ms. Then, a preparatory cue was displayed, allowing the participant to prepare his movement. After a random delay of 1,000 to 1,200 ms, the imperative signal appeared and remained visible until a finger response was detected (700 ms max). We purposely varied the duration of the delay to decrease the tendency to respond prematurely (i.e., before the imperative signal). For the same reason, each block involved some trials in which the bridge did not appear (i.e., catch trials—6 per block), for which the participant was required not to respond. Finally, a feedback score appeared for 500 ms: correct responses led to positive scores (inversely proportional to RT, ceiling at +100), while a fixed negative score (−75) was provided for incorrected responses. The inter-trial interval was set at 2,300 ms.

During each session, the patient performed four blocks of 66 trials during which single-pulse TMS (at 120% of the rMT for each hemisphere) was applied over the left or the right M1 (separate blocks) to elicit MEPs in the contralateral hand. The TMS pulse could be delivered at one of two possible timings (Figure 2C). To establish a baseline measure of corticospinal excitability, the TMS pulse occurred at the onset of the blank screen, eliciting MEPs at rest (TMSBASELINE; 20 MEPs/block). In other trials, TMS was delivered 950 ms after the onset of the preparatory cue, when the response was being withheld (TMSDELAY; 40 MEPs/block). At the latter timing, MEPs could either occur in a hand cued for the forthcoming response (selected condition; e.g. MEPLEFT in left response trials; 20 MEPs/block) or in the non-cued hand (non-selected condition; e.g. MEPRIGHT in left response trial; 20 MEPs/block). The remaining trials (6/block) did not include any TMS pulse, preventing the subject from anticipating TMS pulses at TMSDELAY when it had not occurred at TMSBASELINE. Then, each MEP elicited at TMSDELAY was expressed in percentage of the mean amplitude of MEPs elicited at TMSBASELINE in the corresponding condition (i.e., same muscle and same session); a value below 100 reflects MEP suppression, while a value above 100 indicates MEP facilitation.

#### 2.3.3 TMS protocol

Single- and paired-pulse TMS (biphasic pulses, posterior/anterior—anterior/posterior current direction) were delivered through a 75 mm figure-of-eight coil (C-B60, MagVenture, Denmark) connected to a MagPro X100 magnetic stimulator (MagVenture, Denmark). The coil was placed tangentially on the scalp over the left or right M1 with the handle pointing backwards and laterally at a 45° angle away from the midline, approximately perpendicular to the central sulcus. For each M1, the optimal coil placement for eliciting MEPs in the contralateral first dorsal interosseous (FDI) was identified and marked on a head cap placed on the participant’s scalp to provide a reference mark throughout the experiment (73). The resting motor threshold (rMT) was determined as the minimal TMS intensity required to evoke MEPs of 50 μV peak-to-peak in the relaxed FDI muscle in 5 out of 10 consecutive stimulations. Across the four sessions, the rMT remained steady, as it corresponded to 47, 47, 47 and 46% and to 44, 45, 44 and 42% of the maximum stimulator output for the left and right M1, respectively. Note that because finger representations have a large degree of overlap in M1 (74), TMS pulses applied over the FDI hotspot can also elicit reliable MEPs in other finger muscles. So, during the rolling ball task, MEPs were also recorded in the abductor pollicis brevis (APB) of both hands, as successfully done in past studies (45, 57, 75), which allowed us to obtain measures of corticospinal excitability in a task-irrelevant muscle.

#### 2.3.4 EMG recording

EMG activity was recorded from surface electrodes (Ambu Blue Sensor NF-50-K Neuroline, Medicotest, Oelstykke, Denmark) placed over the FDI and APB muscles of both hands. EMG data were collected for 3,200 ms on each trial, starting always 200 ms before the TMS pulse. The raw EMG signals were amplified (gain, 1 K), bandpass filtered online (10–500 Hz, Neurolog; Digitimer) and digitized at 2,000 Hz for offline analysis. The latter consisted in extracting the peak-to-peak amplitude of MEPs recorded in the FDI, as well as in the-task irrelevant APB during the rolling ball task. In order to prevent contamination of MEP measurements by significant fluctuations in background EMG, trials with visible EMG activity prior to the TMS pulse were removed (76). Moreover, during the task, trials in which the patient had provided the wrong response were also discarded; the task was so easy that these trials remained rare and errors were not analyzed. Following data cleaning, a mean ± SD of 10.8 ± 3.59, 17.5 ± 3.69, 15.3 ± 4.72 and 18.0 ± 4.0 MEPs per condition remained to assess intracortical circuits across the four sessions, while a mean ± SD of 34.7 ± 3.28, 33.9 ± 1.68, 32.3 ± 3.51 and 37.4 ± 2.50 MEPs per condition remained to evaluate changes in motor excitability during action preparation in the corresponding conditions.

### 2.4 Statistical analyses

Motor excitability at rest. In order to evaluate the strength of intracortical circuits across the four TMS sessions, three separate analyses of variance (ANOVAs) were performed on conditioned MEPs (expressed in percentage of changes relative to the mean amplitude of unconditioned MEPs in the corresponding condition) elicited in the SICI, LICI and ICF conditions, using the factor SESSION (1 to 4).

Motor excitability during action preparation. Regarding the rolling ball task, we first analyzed behavior by performing a three-way ANOVA on RTs, including the factors SESSION (1 to 4), TMS-TIMING (TMSBASELINE, TMSDELAY) and RESPONDING-SIDE (left, right). Second, we considered MEPs elicited at TMSDELAY (expressed in percentage of the mean amplitude of MEPs elicited at TMSBASELINE in the corresponding condition), probed both in the FDIs and the APBs. Those MEPs were analyzed using two separate three-way ANOVAs (one for each muscle), with the factors SESSION (1 to 4), CONDITION (selected, non-selected) and RESPONDING-SIDE (left, right). Finally, in order to compare behavior and preparatory changes in corticospinal excitability observed in the patient with data reported in healthy controls, multiple *t*-tests (two-side) were carried out (one for each sub-condition), using the Crawford and Howell’s single case method (77, 78).

With the exception of the *t*-tests, which were performed with the SINGLIMS program (available at https://homepages.abdn.ac.uk/j.crawford/pages/dept/SingleCaseMethodology.htm), all analyses were carried out using Statistica 10 (StatSoft, Cracow, Poland). ANOVAs were followed by post-hoc tests using the Fisher’s least significant difference (LSD) procedure. Please note that, due to an unequal number of trials in each sub-condition preventing us from performing repeated measures ANOVAs, all the within-subject variables were treated as between-subject factors. All data are expressed as mean ± standard error (SE). The statistical significance was set at *p* < 0.05.

## 3 Results

### 3.1 Motor excitability at rest

**Figure 3:**
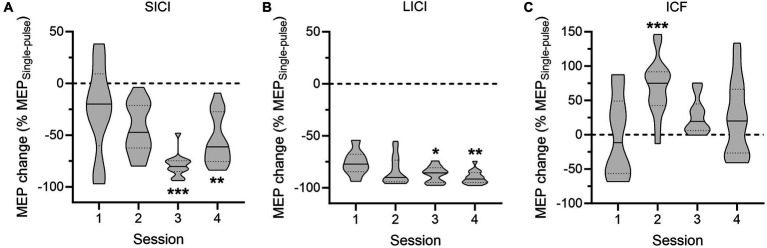
Intracortical circuits probed in the patient. The data represent the amplitude of conditioned motor-evoked potentials (MEPs), expressed in percentage of changes relative to the mean amplitude of unconditioned MEPs, for the short-interval intracortical inhibition (SICI, **A**), long-interval intracortical inhibition (LICI, **B**) and intracortical facilitation (ICF, **C**) conditions across the four sessions. ^*^p < 0.05, ^**^p < 0.01, and ^***^p < 0.001: significantly different from Session 1.

As evidenced on Figure 3, the strength of SICI, LICI and ICF varied across the sessions. Accordingly, the ANOVAs performed on conditioned MEPs (expressed in percentage of unconditioned MEPs) revealed a main effect of the factor SESSION for each condition [*F*(3,50) = 8.69; *p* < 0.001 for SICI; *F*(3,47) = 3.78; *p* < 0.05 for LICI; *F*(3,52) = 5.46; *p* < 0.01 for ICF]. Post-hoc tests run on the SICI condition indicated that MEPs recorded during Sessions 3 and 4 were significantly more reduced relative to MEPs probed during the first session (both *p* < 0.01), with SICI during Session 3 being more pronounced than in all other sessions (all *p* < 0.05). A similar strengthening of intracortical inhibition was observed regarding LICI, as MEPs recorded in the two last sessions were also more suppressed than MEPs probed at Session 1 (both *p* < 0.05). Finally, a different pattern of changes was reported for the ICF condition; here, the facilitation was larger in Session 2 in comparison to the other sessions (all *p* < 0.05).

### 3.2 Motor excitability during action preparation

#### 3.2.1 Behavioral data

**Figure 4:**
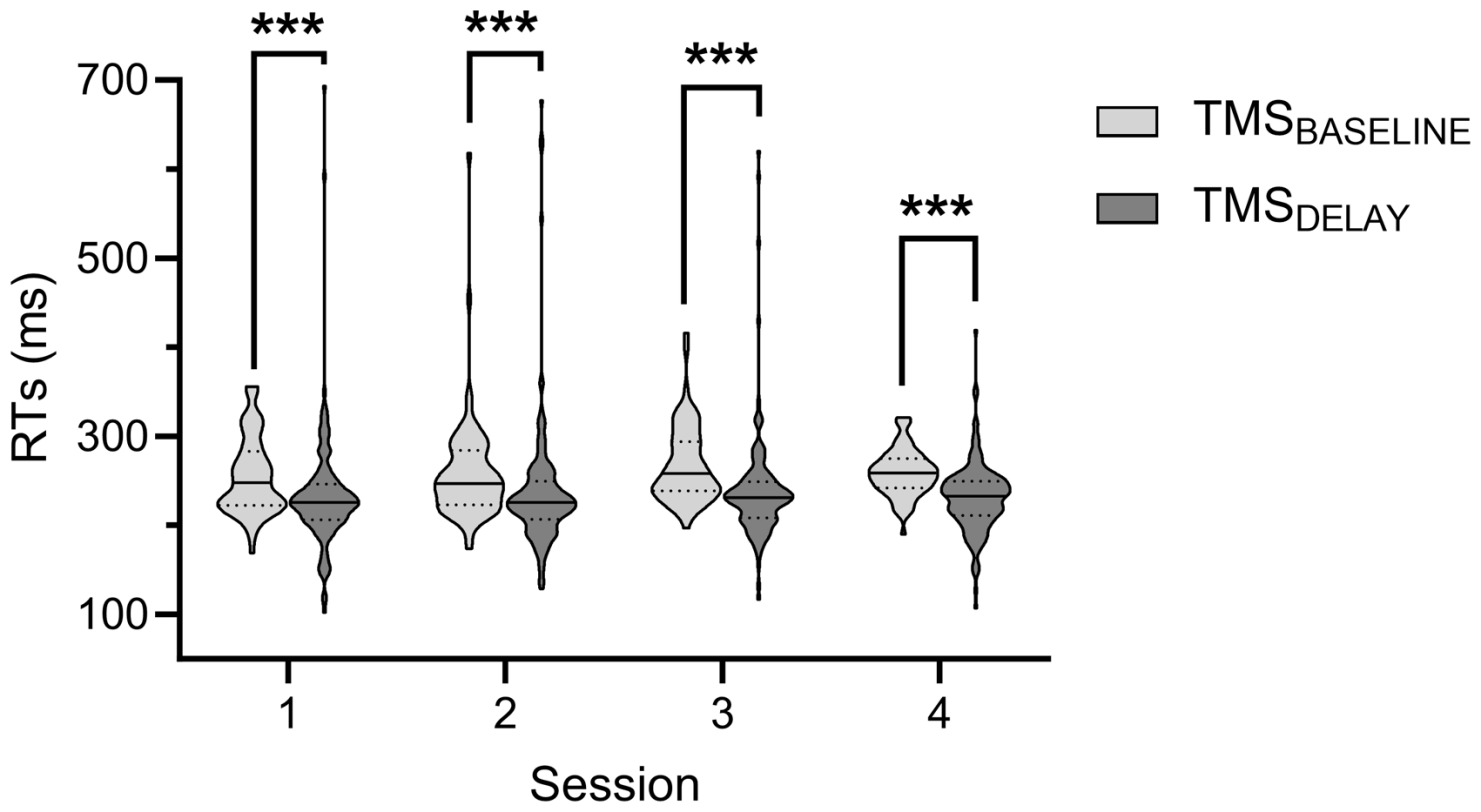
Reaction times (RTs) during the rolling ball task in the patient. The RTs during the four sessions are shown for trials in which the TMS pulse was applied either at baseline (TMS_BASELINE_, light grey) or during action preparation (TMS_DELAY_, dark grey). Data from responses performed with both hands were comparable and thus pooled together. ^***^p < 0.001: significantly different, such as indicated by the main effect of the factor TMS-TIMING.

The RTs measured during the rolling ball task are shown in Figure 4. Analyses revealed a significant effect of TMS-TIMING [*F*(1,812) = 32.57; *p* < 0.001]: RTs were shorter at TMSDELAY than at TMSBASELINE, consistent with many reports showing that a TMS pulse applied close to the imperative signal can speed up the release of a motor response (46, 79). Besides, neither the factor SESSION [*F*(3,812) = 1.11; *p* = 0.34], nor the factor RESPONDING-SIDE [*F*(1,812) = 3.75; *p* = 0.06] or any of the interactions were significant (all *F* < 1.13 and all *p* > 0.28). In controls, the RTs equaled 286.2 ± 7.69 and 273.2 ± 6.80 ms for responses performed at TMSBASELINE and TMSDELAY, respectively. While a comparison of these numbers to RTs displayed on the figure seemed to indicate that the patient might be faster than controls, the difference was not significant (both |*t*| < 0.82 and both *p* > 0.21).

#### 3.2.2 MEP data

**Figure 5:**
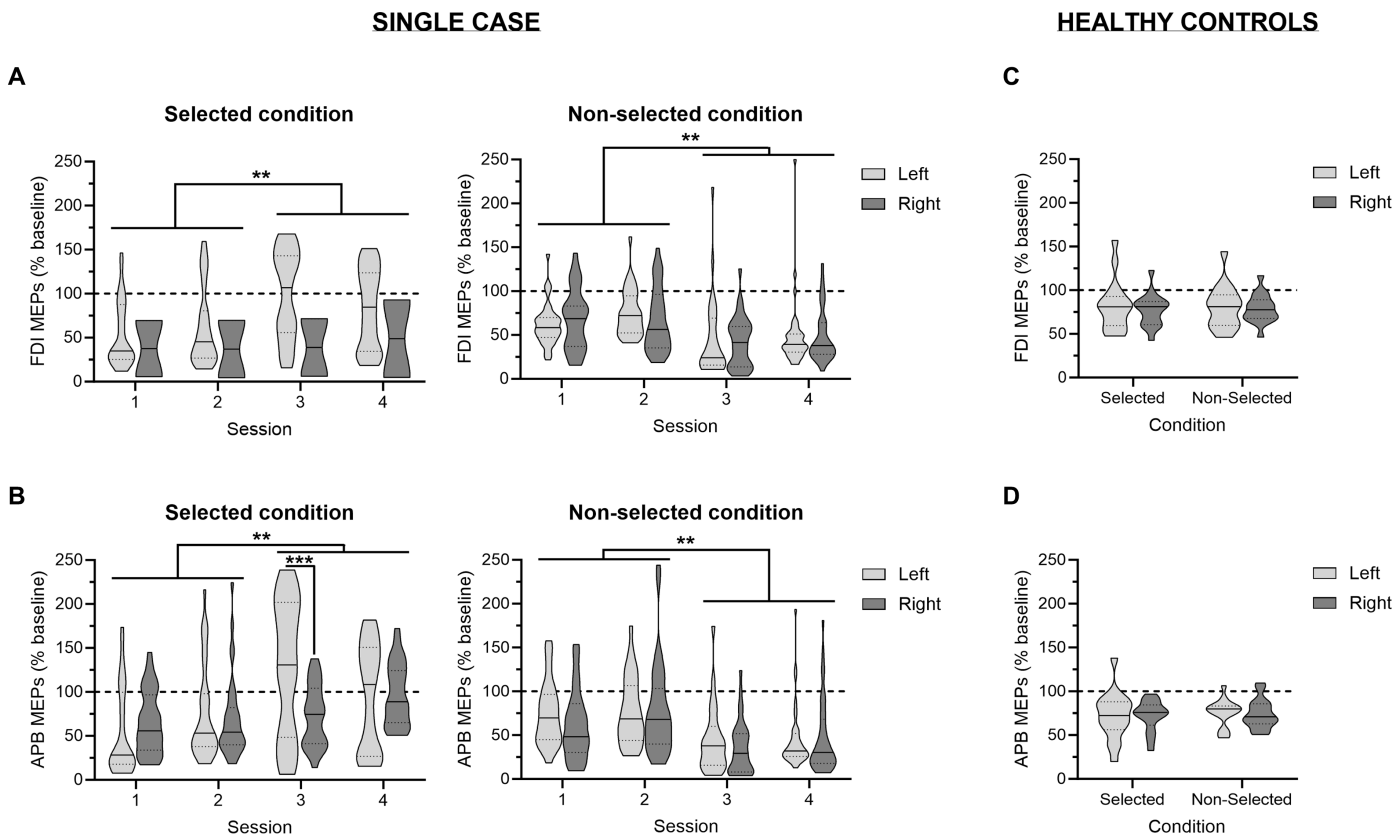
Changes in corticospinal excitability during action preparation. Amplitudes of motor-evoked potentials (MEPs) recorded at TMS_DELAY_, expressed in percentage of MEPs elicited at TMS_BASELINE_, are displayed for the patient **(A,B)** and for a reference group of healthy controls from a previous study **(C,D)** (45); in the task-relevant first dorsal interosseous (FDI; **A,C**) and in the task-irrelevant abductor pollicis brevis (APB; **B,D**) for trials in which responses were performed with the left (light grey) or the right (dark grey) hand. The data are depicted separately for the selected (left panel) and non-selected (right panel) condition. For the patient, data are shown across the four sessions. ^**^p < 0.01: significantly different, such as indicated by the significant SESSION × CONDITION interaction. ^***^p < 0.001: significant difference between left- and right-hand trials.

A glimpse at Figure 5A provides a global picture of preparatory changes in corticospinal excitability observed in the FDI—i.e., the task-relevant muscle—across the four sessions. As can be seen by comparing the left and the right panel of the figure, analyses performed on MEPs probed at TMSDELAY (expressed in percentage of MEPs probed at TMSBASELINE) revealed a main effect of the factor CONDITION [*F*(1,534) = 34.89; *p* < 0.001], due to a stronger suppression of MEPs in the non-selected relative to the selected condition. Interestingly, whereas the factor SESSION was not significant [*F*(3,534) = 0.66; *p* = 0.57], there was a significant interaction between these two variables [SESSION × CONDITION interaction; *F*(3,534) = 18.08; *p* < 0.001], indicating that changes in corticospinal excitability evolved differently across the four sessions depending on the condition. As such, MEPs probed in the selected FDI were greater during the two last sessions relative to the two first ones (all *p* < 0.01), while there was no significant difference between Sessions 1 and 2 (*p* = 0.61) and Sessions 3 and 4 (*p* = 0.81). By contrast, MEPs elicited in the non-selected FDI displayed a diametrically reversed pattern of changes; here, MEPs probed during Sessions 3 and 4 were significantly smaller than MEPs recorded during Sessions 1 and 2 (all *p* < 0.01), whereas MEPs elicited during the two first (*p* = 0.31) and the two last sessions (*p* = 0.44) were not significantly different. In other words, those results revealed that the strength of preparatory changes in corticospinal excitability was modified once the symptomatology of the patient had improved, with this change appearing in the form of enhanced excitatory and inhibitory influences. Finally, analyses showed a significant SESSION × RESPONDING-SIDE interaction [*F*(3,534) = 3.30; *p* < 0.05], which was explained by larger MEPs when responses involved the left rather than the right-hand during Session 3 (*p* < 0.01), regardless of the condition, while there was no significant difference in the other sessions (all *p* > 0.09). No other main effect or interaction was significant (all *F* < 2.13 and all *p* > 0.09).

A similar pattern of changes was observed in the task-irrelevant muscle; i.e. the APB (Figure 5B). As such, a significant main effect of the factor CONDITION [*F*(1,514) = 37.27; *p* < 0.001] as well as a significant SESSION × CONDITION interaction [*F*(3,514) = 21.13; *p* < 0.001] were also observed. However, here, the evolution of preparatory changes in the selected and the non-selected hand across the four sessions depended on the responding-side [SESSION × CONDITION × RESPONDING-SIDE interaction; *F*(3,534) = 3.47; *p* < 0.05]. Hence, in the selected condition, the increase in MEPs across the sessions was already manifest at Session 2 for responses performed with the left hand (i.e., Session 2 vs. Session 1; *p* < 0.05), while it only appeared during the last session when responses involved the right hand (i.e., Session 4 vs. Sessions 1–3; all *p* < 0.05). As a result, there was a significant difference between MEPs probed in APB of the selected hand for responses performed with the left and the right hand at Session 3 (*p* < 0.001). By contrast, in the non-selected condition, the strengthening of inhibitory influences during the two last sessions relative to the two first ones did not depend on the responding side, similarly to what was observed in the FDI.

Figures 5C,D display the changes in corticospinal excitability during action preparation probed in healthy controls. Such as previously discussed in the original paper (45), those MEPs were significantly reduced relative to MEPs elicited at TMSBASELINE. This effect concerned both task-relevant and task-irrelevant muscles, and occurred regardless of the condition and the responding side. Interestingly, the *t*-tests comparing MEPs recorded in the patient to values obtained in controls did not reveal any significant difference, indicating that he did not suffer from an alteration in the dynamics of corticospinal excitability changes during action preparation. If anything, the patient tended to display stronger inhibitory influences than controls, especially in the two last sessions [e.g., *t*(15) = −1.92 and *p* = 0.07 or *t*(15) = −1.85 and *p* = 0.08 for MEPs probed in the APB of the non-selected hand during right hand trials in Sessions 3 and 4, respectively]. A tendency for a higher excitatory drive was also observed, notably in the selected APB during left hand trials in Session 3 [*t*(15) = 2.01; *p* = 0.06].

## 4 Discussion

OCD, even more than GTS, is a neurodevelopmental disorder that has been associated with a loss of inhibitory control at a behavioral level (30, 40, 41). Here, we aimed at focusing on motor inhibition at a physiological level by assessing intracortical M1 circuits at rest and by considering the strength of motor suppression during action preparation, as a fingerprint of inhibitory processes involved in the regulation of movement initiation (42, 58, 59). In order to do so, the present study used TMS over M1 to measure motor intracortical circuits at rest as well as changes in corticospinal excitability during action preparation in a 25 years-old patient that presented with both a GTS and a severe OCD, where impairments in motor control were also central. Importantly, the patient performed four experimental sessions from February to April 2021, a period during which his symptomatology drastically improved, coinciding with feedbacks of good motor control abilities that were given to the patient. We evaluated whether these behavioral changes were reflected in the TMS measurements.

We had the opportunity to test the patient for the first time during one of his stays in the psychiatric department of CUSL, in an attempt to identify potential alterations in motor excitability that might guide future treatment. Accordingly, paired-pulse TMS was used to measure intracortical inhibitory and facilitatory circuits within M1. While we did not include data from healthy controls for this part of the protocol, our results tend to indicate that the patient suffered from a lack of intracortical inhibition, especially manifest in the SICI condition. As such, while the amplitudes of conditioned MEPs are usually reduced by 50% to 90% in SICI paradigms when probed in healthy subjects (20, 80, 81), MEPs in the patient were only suppressed by 25% during the first testing. The fact that SICI reached a normal range in the later sessions also indirectly supported the presence of a deficit in the first sessions. Besides, although less obvious in this condition, the strengthening of LICI in the two last sessions also suggests that it was slightly deficient at Session 1. Overall, this lack of intracortical inhibition is in line with the alteration of GABAergic systems previously identified in OCD and GTS (17, 18, 24), as well as with TMS data demonstrating deficient SICI at rest, again both in OCD and GTS (20–23, 25–27, 82), although findings have not been consistent in all studies (28, 29). Finally, results were less clear regarding the ICF condition; here, we observed a lack of changes in conditioned MEPs in Session 1 followed by a visible facilitation during Session 2. Interestingly, this inconsistency also characterizes prior works, in which ICF was found to be either enhanced (83, 84) or normal (20, 21) in GTS patients, an effect that could simply be explained by the well-recognized variability in ICF measurements (85).

By contrast, MEPs probed during action preparation in Session 1 in the patient were similar to preparatory changes observed in healthy controls, both in task-relevant (FDI) and task-irrelevant (APB) muscles. That is, MEPs were reduced relative to resting conditions, indicating a strong suppression of corticospinal excitability in the patient, consistently reported in healthy subjects (47, 72, 79, 86). As mentioned earlier, this neural suppression of motor excitability during action preparation has been related to some sort of inhibitory control allowing to avoid premature responding during action preparation (42, 43, 58), even if there are also alternative views though not necessarily incompatible [(48, 51) for an alternative view]. Our interpretation of rather good inhibitory control in the patient (based on the observation of normal preparatory suppression) is in line with growing evidence indicating normal inhibitory functions in GTS. As such, it is becoming clear from the literature that GTS patients are able to suppress or cancel inappropriate motor responses in Go/No-Go and Stop-signal tasks, possibly due to the constant need to suppress tics, leading to efficient inhibitory control of motor outputs (30, 36, 40, 41, 87–89). Yet, the most prominent symptoms of our patient were those of OCD, a condition which has been associated with more inhibitory deficits in the same tasks (30, 40, 41). One plausible explanation is that the MEP suppression we observe during action preparation is not a reflection of the type of inhibitory control recruited in stop signal tasks, as already suggested by ourselves in a past study on alcohol-dependent patients (58) and as also consistent with a recent reflection on the complexity of constructs such as inhibitory control and impulsivity that are often related but that do clearly not overlap (31). Clearly, the mechanisms underlying the inability to control urges in psychiatric disorders are extremely heterogeneous and cannot be ascribed to a general impairment of motor inhibition. Moreover, the different types of tasks that are used in the field are likely to tap into separate facets of inhibitory control and thus non-surprisingly often provide different outcomes (30). Thus, the present study adds to this literature and supports the idea that the constant need to suppress tics leads to efficient inhibitory control of motor outputs during movement preparation, despite a loss of motor intracortical inhibition at rest (34, 35) and, as observed with this patient, a lower self-efficacy feeling regarding own inhibition abilities (90).

To summarize, we have shown that when tested for the first time, the patient likely suffered from a lack of intracortical inhibition within M1 (coming along with a weak self-efficacy feeling), while the suppression of corticospinal excitability during action preparation was in fact normal. Interestingly, past studies have already highlighted a dissociation between the level of inhibition probed at rest and during a task. For example, by investigating both aspects, Heise et al. (20) showed that SICI at rest was drastically less pronounced in GTS patients relative to controls, whereas it rapidly increased until reaching normal values during movement preparation. Similarly, motor excitability was found to be lower in patients asked to control their tics relative to a resting condition (32). Those results can be interpreted as reflecting compensatory mechanisms implemented to establish adequate online behavior control (20). Such as developed by the authors, during the resting state, motor excitability would be highly influenced by abnormally active afferent inputs, and consequently characterized by a loss of intracortical inhibition. By contrast, when some control is required, motor excitability would be down-regulated to allow the system to generate an appropriate motor response, including the suppression of tics. Finally, note that a similar dissociation has been observed at the behavioral level (91): while automatic inhibition, a process not submitted to voluntary control, was impaired in GTS patients, volitional reactive and proactive inhibition was normal in the same individuals of that study, indicating that the lack of inhibition in GTS specifically concerns situations in which control is not required.

Strikingly, the pattern of results described above was remarkably similar during the two first sessions, with the exception of ICF which is known to be more variable (85). At that time, the patient reported significant clinical suffering. He was spending all the time in his room during his stays in the hospital, sitting most of the day on a chair, unable to dress or undress and to wash, presenting with episodes of loss of bladder control, and spending most of the time in compulsions and obsessive thoughts, taken up by frequent episodes of tics without any significant evolution between the two sessions. Hence, levels of intracortical inhibition and preparatory changes remained stable at a time during which the symptomatology did not evolve. At the end of the second session, CQ had a long discussion with the patient, aiming at giving him some feedback. In particular, she insisted on the fact that he had the appropriate cognitive resources when required, to exert control over his behavior. Outstandingly, the patient drastically changed following this discussion: he decided to quit his room to participate to the activities of the group of patients, he was suddenly able to interfere with, willing to establish social contacts and after the activities, proposing to play table tennis. A new mobility emerged concerning entering and going out of his room; he started washing and dressing and obsessions and compulsions were still present but with a lower intensity. The control of the bladder was still unperfect. We interpreted this dramatic change in behavior as a consequence of a regained confidence in his abilities for motor (and thought) control, following the discussion with CQ. Most fascinatingly, those improvements occurred simultaneously with considerable changes in MEP measurements of Sessions 3 and 4. As such, SICI and LICI were more pronounced than previously, revealing a strengthening of intracortical inhibition. Moreover, MEPs probed during action preparation evolved in opposite directions depending on whether they were elicited in the selected or non-selected hand for the forthcoming response. As such, whereas MEPs increased in the selected condition, their amplitude decreased even more in the non-selected one, possibly reflecting enhanced excitatory and inhibitory influences, respectively: the patient had recovered the ability to control selective excitation and inhibition of movement, as evidenced in the selected and non-selected hands. Finally, the pattern of MEP changes stayed relatively constant in Sessions 3 and 4.

Interestingly, the strength of SICI has been related to OCD symptoms (26, 82) and to tic severity (22, 23), suggesting that its strengthening could have been related to the disappearance of obsessive thoughts and tics in the patient. Regarding our findings during action preparation, we believe that they reflect an increased tendency to act (lower corticospinal suppression in selected hand), associated with stronger inhibitory influences during action preparation (deeper MEP suppression in the non-selected hand), even though the latter already fell in a normal range at the beginning of the study. Interestingly, these changes came along with a recovery of self-efficacy, i.e., the individual’s beliefs in the capacity to act in the way necessary to reach specific goals, that is currently considered as central for the improvement of symptoms expression in psychopathology (90). Importantly, MEPs induced by single-pulse TMS represent global readouts of motor excitability and their amplitude is influenced by multiple excitatory and inhibitory processes acting at the same time (39, 92). On the one hand, MEPs probed in the selected condition depend on inhibitory processes allowing to withhold the response until the appropriate time, but also on excitatory influences dedicated to prepare the system for the forthcoming action. As the latter can drastically increase when the readiness to initiate actions is enhanced (59, 61, 93, 94), the larger MEPs observed in Sessions 3 and 4 could be attributed to the stronger motivation—or diminished apathy—reported by the patient. On the other hand, MEPs elicited in the non-selected condition are spared from this excitatory drive, and consequently more purely reflect inhibitory influences associated with the suppression of inappropriate responses. Hence, the larger MEP suppression observed here would indicate improved inhibitory control in the patient. The changes in MEPs over the sessions could also potentially reflect recovered confidence and self-efficacy of the patient. Finally, as changes in intracortical and corticospinal preparatory activity occurred in parallel, we might suggest that both improvements were related. In line with this assumption, the level of resting SICI predicts response inhibition abilities in healthy subjects (95), while improved inhibitory control following a stop-signal training correlates with increases in SICI strength at rest (96). Moreover, between-group differences in the level of resting SICI persists when evaluated during inhibitory tasks (97, 98). Consequently, we might suppose that the strengthening of SICI observed in the patient was associated with his increased inhibitory abilities. Nevertheless, as SICI was not measured during the rolling ball task, this question remains unclear.

Finally, some hypotheses can be put forward regarding the neural mechanisms contributing to MEP changes during the two last sessions. In particular, we posit that they would result from a better ability of the patient, once he had gained confidence in his cognitive resources, to implement further compensatory self-regulation mechanisms. This notion of compensatory mechanisms in GTS has been already proposed to explain the gradual decrease in the frequency and intensity of tics throughout adolescence (2, 35). According to this idea, individuals would gain control over motor outputs through structural and functional changes in neural pathways linking prefrontal and motor regions (34, 88, 99–102). Importantly, MEPs probed during action preparation are modulated by inputs originated from upstream cognitive regions (42, 43). As such, the level of suppression seems to be related to pre-supplementary motor area and to prefrontal, premotor and anterior cingulate cortices (56, 103, 104). Therefore, it might be that the patient became able to recruit more frontal regions during the task, leading to a stronger suppression of motor excitability during action preparation. Finally, the strengthening of SICI and LICI could be attributed to an increase of the inhibitory tone in motor areas (35, 105). While this could be due to a compensatory reinforcement of tonic inhibition, we cannot exclude that the patient was simply in a higher control state, even during resting measures.

In conclusion, by repeatedly evaluating a patient suffering from OCD and GTS, the present study shows that motor inhibition probed at a physiological level, both at rest and during action preparation, can rapidly increase, along with a greater tendency to act, and that these changes can serve as valuable indicators of improved clinical expression. More importantly, reassuring the patient about his cognitive resources, based on objective brain measures, might have considerable beneficial effects. In the future, providing OCD/GTS patients with this type of biofeedback could be implemented as a novel treatment approach to improve mechanisms related to inhibition and inhibitory control, and consequently their symptomatology. Ideally, these future studies should also involve measures of inhibitory control at a behavioral level, for an even more detailed analysis of the patient progress accompanying the biofeedback intervention.

## Data availability statement

The raw data supporting the conclusions of this article will be made available by the authors, without undue reservation.

## Ethics statement

The studies involving humans were approved by Biomedical Ethics Committee of the Saint-Luc University Hospital. The studies were conducted in accordance with the local legislation and institutional requirements. The participants provided their written informed consent to participate in this study. Written informed consent was obtained from the individual(s) for the publication of any potentially identifiable images or data included in this article.

## Author contributions

CQ: Conceptualization, Formal analysis, Investigation, Methodology, Writing – original draft, Writing – review & editing. FC: Writing – review & editing. JD: Conceptualization, Funding acquisition, Methodology, Supervision, Writing – review & editing. PT: Conceptualization, Funding acquisition, Methodology, Writing – original draft, Writing – review & editing.
